# Trials to Overcome Drug Resistance to EGFR and ALK Targeted Therapies – Past, Present, and Future

**DOI:** 10.3389/fonc.2014.00233

**Published:** 2014-08-27

**Authors:** Johanna N. Spaans, Glenwood D. Goss

**Affiliations:** ^1^Ottawa Hospital Research Institute, Ottawa, ON, Canada; ^2^Ottawa Hospital Cancer Centre, Ottawa, ON, Canada; ^3^Department of Medicine, University of Ottawa, Ottawa, ON, Canada

**Keywords:** EGFR, ALK, resistance, molecular therapy, clinical trial

## Abstract

Molecularly targeted agents are changing the therapeutic landscape in advanced non-small cell lung cancer. Since the discovery of sensitizing mutations in the epidermal growth factor receptor (EGFR) and anaplastic lymphoma kinase (ALK) domain, clinical investigations have focused on optimizing the efficacy of EGFR and ALK tyrosine kinase inhibitors by addressing therapeutic resistance that commonly develops within a year of treatment initiation. Here, we review the clinical trials of novel therapies and combination regimens that have been undertaken in response to our evolving understanding of the mechanisms of resistance to targeted therapy. The aim of these trials was to enhance the therapeutic efficacy of targeted therapies by improving blockade and/or inhibiting parallel or compensatory signaling pathways. We have documented the sequential conduct of EGFR and ALK biomarker-driven trials in order to highlight particular pitfalls and successes, which should be considered in the design of future trials. Although there remain significant challenges, substantial gains have been made in our understanding of cellular resistance. This knowledge will drive the design of future trials to the benefit of lung cancer patients.

Lung cancer is the leading cause of cancer related deaths worldwide (1). Non-small cell lung cancer (NSCLC) represents approximately 85% of all lung cancers (2). In the advanced disease setting, systemic platinum-based chemotherapy yields survival rates of approximately 1 year (3). In the last decade, the targeted inhibition of oncogenic driver mutations with molecular therapies of which the epidermal growth factor receptor (EGFR) and the anaplastic lymphoma kinase (ALK) are the most studied targets, has seen dramatic improvements in overall survival in defined subsets of patients (4, 5). However, despite impressive early response rates, most patients progress within a year (4). Herein, we review clinical trials undertaken in response to our evolving understanding of the oncogenic drivers and molecular mechanisms of drug resistance.

## Background

Given its important role in tumor growth and proliferation, the EGFR pathway has been the focus of intense clinical investigation across many tumor sites (6, 7). The high protein expression levels observed in NSCLC across all histologies (8), particularly among those with advanced disease (9), provided the initial impetus for the early lung cancer trials targeting the EGFR pathway by small molecule tyrosine kinase inhibitors (EGFR-TKIs) and anti-EGFR monoclonal antibodies.

Early trials in advanced NSCLC evaluated EGFR-TKIs as both monotherapy after chemotherapy failure and in combination with chemotherapy in the first-line setting. As a monotherapy, the EGFR-TKI erlotinib, was shown to improve progression free survival (PFS) (2.2 vs. 1.8 months, *p* < 0.001) and overall survival (OS) over best supportive care (6.7 vs. 4.7, *p* < 0.001) in unselected NSCLC patients with advanced disease who had failed one or two prior lines of chemotherapy (BR 21) (10). Although gefitinib, another EGFR-TKI, was similarly able to delay disease progression over placebo in the second/third-line setting (3.0 vs. 2.6 months, *p* = 0.0006), the lack of overall survival benefit (5.6 vs. 5.1 months, *p* = 0.087) in the definitive phase III trial (ISEL) (11)resulted in the withdrawal of gefitinib’s accelerated FDA approval, which was based on encouraging phase II data (IDEAL-1, IDEAL-2) (12, 13). Disappointingly, when used in combination with upfront chemotherapy in unselected patients with advanced NSCLC, neither erlotinib nor gefitinib was shown to improve overall survival (14–17). The strategy of combining EGFR-TKIs and chemotherapy, therefore, has since largely been abandoned.

Objective responses in most of the early EGFR-TKI trials in unselected patients was quite variable, with many trials independently identifying a small subgroup of extreme responders (10, 11), against a large background of patients with primary resistance to EGFR inhibition. In these trials, the extreme responders were most commonly defined by their clinical and ethnic characteristics (Asian, non-smokers) and not by their pre-treatment EGFR protein expression levels. With the discovery of the activating EGFR mutations in exon 19 and 21 of the kinase domain (18, 19), came a number of retrospective mutational studies of the earlier anti-EGFR trials, which confirmed the importance of these mutations (20, 21). These studies lead to the molecular characterization of EGFR-TKI responders and the subsequent EGFR mutation-positive biomarker-driven trials, detailed below.

## Past Clinical Trials Addressing Resistance

The restriction of EGFR-TKI trials to patients whose tumors harbored activating EGFR mutations represented the first attempt to address primary drug resistance, by limiting exposure in patients unlikely to benefit from EGFR-TKIs. Six large randomized phase III trials that enriched or selectively enrolled patients with activating EGFR mutations definitively confirmed the benefit of first-generation reversible EGFR-TKIs over standard chemotherapy in the first-line setting (Table 1) (22–27). These trials, which collectively enrolled over 2200 patients, showed a doubling of response rate to 60–80% over chemotherapy alone in patients whose tumors harbored the EGFR mutation (22–27). In the four trials that only enrolled EGFR-mutant positive NSCLC patients (two gefitinib, two erlonitib), EGFR-TKIs were shown to extend PFS by 3–8 months (24–27). Consequently, targeted monotherapy with erlotinib or gefitinib has now become standard of care in the first-line setting in patients with EGFR mutation-positive tumors.

**Table 1:** **Randomized phase III trials of first-generation EGFR-TKIs in EGFR mutation (+)/enriched populations**.

Study	Agent	EGFR+/N	PFS (EGFR+) (months)	OS (EGFR+) (months)
NEJ002 (24)	Gefitinib	224/224	10.8 vs. 5.4 (HR 0.3)	27.7 vs. 26.6 (HR 0.89)
WJTOG-3405 (25)	Gefitinib	192/192	9.2 vs. 6.3 (HR 0.5)	36 vs. 39 (HR 1.19)
OPTIMAL (26)	Erlotinib	154/154	13.1 vs. 4.6 HR 0.16	HR 1.065
EURTAC (27)	Erlotinib	153/153	9.7 vs. 5.2 HR 0.37	19.3 vs. 19.5 HR 1.04
IPASS (22)	Gefitinib	261/1217	9.5 vs. 6.3 HR 0.48	21.6 vs. 21.9 HR 1.0
SIGNAL (23)	Gefitinib	42/309	8.0 vs. 6.3 HR 0.54	27.2 vs. 25.6 HR 1.04

Due to their different mechanism of action, anti-EGFR monoclonal antibodies have also been evaluated in the management of advanced NSCLC. The most well studied of these agents is cetuximab, a monoclonal chimeric IgG1 antibody, that inhibits EGFR pathway activation by binding to the EGF receptor (28). Unlike its small molecule counterpart, monotherapy trials of cetuximab were disappointing in advanced NSCLC (29). However, cetuximab has been successfully combined with chemotherapy in the first-line setting. In a large phase III trial that enrolled 1125 patients with advanced NSCLC (FLEX) (30), cetuximab was shown to improve overall survival (11.3 vs. 10.1 months, *p* = 0.004) when combined with cisplatin and vinorelbine, with greater efficacy noted in patients with higher EGFR protein expression (31). While a similar smaller study (*n* = 676) of cetuximab with a carboplatin/paclitaxel regimen (BMS099) (32) did not demonstrate improved PFS or OS with the addition of cetuximab, a meta-analysis of four randomized phase II&III trials (which included the BMS099 study results) did show that cetuximab with first-line platinum-based chemotherapy improved both PFS and OS (33). Cetuximab’s inconsistent and limited clinical efficacy, however, has restricted the uptake and regulatory approval of this anti-EGFR monoclonal antibody in advanced NSCLC. While other anti-EGFR monoclonal IgG1 antibodies, such as matuzumab, have demonstrated some efficacy in combination with chemotherapy in the second-line setting in phase II trials (34), other anti-EGFR monoclonal IgG2 antibodies (e.g., panitumumab) in combination with chemotherapy have shown little activity, even in patients with EGFR-mutant disease (35). Newer fully human recombinant anti-EGFR IgG1 monoclonal antibodies (e.g., necitumumab), which lack the immunoreactivity of earlier chimeric (human/mouse) monoclonal antibodies, are currently under investigation in certain histological subgroups in combination with chemotherapy, as detailed below.

As noted previously, even in the presence of sensitizing EGFR mutations, only 60–80% of patients with advanced NSCLC respond to EGFR-TKIs. Despite earlier failed attempts to combine EGFR-TKIs with chemotherapy, other combination regimens were evaluated early in the clinical development of EGFR-TKIs, in an attempt to expand and enhance their therapeutic efficacy. To understand the rationale for these, and other combination regimens evaluated in molecular oncology clinical trials, it is useful to consider the therapeutic strategies advanced by Dancey and colleagues (36) to address targeted therapy drug resistance, namely to (1) augment the first agent’s activity; (2) enhance single target blockade; (3) inhibit multiple targets or multiple pathways; and (4) inhibit compensatory pathways. While combination therapies with chemotherapy used the first of these strategies, strategies two to four have guided most clinical research in the acquired resistance setting, as detailed below.

### Past trials of combination therapies

Dual targeting of a single receptor was the rationale behind the early trials of combined EGFR-TKI and anti-EGFR monoclonal antibody therapies, which, due to the off-target effects of the first-generation EGFR-TKIs, proved very toxic (37). Multiple pathway inhibition was the guiding strategy behind combining EGFR-TKIs with the anti-VEGF monoclonal antibody, bevacizumab, given its earlier success in improving overall survival in advanced NSCLC when given with chemotherapy (38). Although early trials of dual VEGF and EGFR inhibition were encouraging (39, 40), more recent trials in unselected populations of VEGF/EGFR inhibitors, such as vandetanib, do not support this approach in the management of advanced NSCLC (41).

## Current Clinical Trials Addressing Resistance

Despite dramatic responses in patients whose tumors harbor EGFR activating mutations, most patients become resistant to EGFR-TKIs within the first year (10, 11). The majority of current trials are, therefore, focused on addressing the mechanisms of acquired resistance (Table 2). The most common of these mechanisms is the development of a second mutation of the EGFR, namely the T790M mutation (T790M), which occurs in up to 60% of those with EGFR-TKI resistant disease (42). The newer second-generation pan-HER irreversible inhibitors provide compensatory pathway inhibition by direct targeting of the resistant T790M mutation and bind irreversibly to 2 or more receptors of the EGFR domain, thus providing enhanced EGFR pathway blockade. Of the second-generation inhibitors, afatinib and dacomitinib have been the most extensively studied, in both heavily pre-treated patients and in the first-line setting. Afatinib has been evaluated in an EGFR-mutant enriched population who had failed one to two lines of chemotherapy and an EGFR-TKI (LUX-Lung 1) (43). In this placebo-controlled phase III trial, afatinib was shown to improve PFS (3.3 vs. 1.1 months, *p* < 0.0001) but not overall survival (10.8 vs. 12.0 months, *p* = 0.87) (43). A similar study was also undertaken with dacomitinib (BR. 26) (44) in patients who had failed one to three lines of chemotherapy and an EGFR-TKI. As with afatinib, dacomitinib showed improved PFS (2.7 vs. 1.4 months, *p* < 0.0001) but did not improve OS (6.8 vs. 6.3 months, *p* = 0.099) (44).

**Table 2:** **Select trials addressing acquired resistance to targeted therapy**.

Line of therapy	Agents	Trial	PFS (months)
**MONOTHERAPY TRIALS TARGETING THE EGFR DOMAIN**			
First line	Afatinib vs. pem/cispl	LuxLung 3 (45)	11.1 vs. 6.9 (*p* = 0.001)
First line	Afatinib vs. gem/cispl	LuxLung 6 (46)	11.0 vs. 5.6 (*p* < 0.0001)
First line	Dacomitinib vs. gefitinib	ARCHER 1050	Results pending
Second line	Afatinib vs. placebo	LuxLung 1 (43)	3.3 vs. 1.1 (*p* < 0.0001)
Second line	Dacomitinib vs. placebo	BR 26 (44)	2.7 vs. 1.4 (*p* < 0.0001)
**COMBINATION THERAPIES TARGETING THE MET COMPENSATORY PATHWAY**			
Second/third line	Tivantinib + erlotinib vs. erlotinib + placebo	Marquee^a^ (47)	3.6 vs. 1.9 (*p* < 0.0001)
Second/third line	Onartuzumab + erlotinib vs. erlotinib + placebo	METLung^a^ (48)	2.7 vs. 2.6 (*p* = 0.92)

*^a^Trial stopped early for futility to meet primary endpoint*.

Unlike the disappointing results observed in the second/third-line setting, second-generation EGFR inhibitors have proven effective in the first-line setting in patients whose tumors harbor EGFR activating mutations. Specifically, afatinib has been shown to improve PFS compared to pemetrexed/cisplatin (LUX-Lung 3) (11.1 vs. 6.9 months, *p* = 0.001) (45) and, more recently, compared to gemcitabine/cisplatin (LUX-Lung 6) (11.0 vs. 5.6 months, *p* < 0.0001) (46). Dacomitinib is also being evaluated in a head-to-head phase III trial against gefitinib (ARCHER 1050), with the results expected next year (www.clinicaltrials.gov: NCT01774721).

In the context of acquired resistance to anti-EGFR therapies, the activation of alternate signaling pathways by both adaptive mutations that develop outside the EGFR kinase domain and mutation-independent factors have also been described (49), and are informing novel combination therapies to address these acquired mechanisms of resistance. The most common of these alterations is MET activation, which occurs in up to 20% of patients with acquired resistance (50). Both small molecule inhibitors targeting MET and anti-MET monoclonal antibodies have been combined with first-generation EGFR-TKIs. The small molecule, tivantinib, and the monoclonal antibody onartuzumab have both been evaluated in the second-line setting in EGFR-TKI naïve patients after chemotherapy failure. In phase II trials, patients were screened and stratified by EGFR mutation status with planned subgroup analysis by both EGFR mutation and pre-treatment tumor MET expression levels. Despite promising phase II data in patients whose tumors were strongly positive for MET by immunohistochemistry (IHC) (51), the combination of onartuzumab and erlotinib in the phase III trial was not shown to improve PFS (2.7 vs. 2.6 months, *p* = 0.92) or objective response rate (ORR) (8.4 vs. 9.6%, *p* = 0.63) and was stopped early for futility to meet its primary endpoint of improved OS (48). The small molecule tivantinib had a similar outcome, with the phase III trial discontinued prematurely for futility for the primary outcome of improved overall survival, which at the planned interim analysis was comparable to EGFR monotherapy (8.5 vs. 7.8, HR = 0.98, *p* = 0.81) (47). Given the failure of these phase III trials to improve survival, direct targeting of MET as a strategy to enhance EGFR-TKI efficacy has an uncertain future.

The failure of dual MET/EGFR inhibition to improve OS in the second-line setting can, in part, be attributed to fact that none of these trials, which were designed to address acquired resistance to EGFR-TKIs, restricted enrollment to patients with tumors harboring EGFR activating mutations and due to the lack of a robust biomarker predictive of efficacy to MET inhibition. The more focused strategy applied in the first-line setting to evaluate of the first and second-generation EGFR-TKIs, and more recently to ALK inhibitors, which limited enrollment to EGFR mutation-positive and ALK mutation-positive NSCLC, respectively, have been far more successful. In the case of the ALK inhibitor, crizotinib, accelerated approval was granted based on clinical activity observed in a phase I dose-escalation and expansion study, which screened over 1500 pre-treated patients and selectively enrolled 82 patients whose tumors screened positive for the ALK rearrangement (5% prevalence) (52). Results of the definitive phase III trial of first-line crizotinib compared to chemotherapy have recently confirmed these initial findings (53). Using a similar approach of selective enrollment, the second-generation ALK inhibitor ceretinib (LDK378) has also recently achieved regulatory FDA drug approval in patients who have failed the crizotinib, by demonstrating antitumor activity and ORR of ~60% in a heavily pre-treated populations in a phase I trial (ASCEND-1) (54).

## The Future of Clinical Trials of Acquired Resistance

Moving forward, clinical trials of acquired resistance will continue to focus on the testing of novel monotherapies and combination therapies targeting the EGFR kinase domain. In the former case, phase III clinical trials are currently on-going comparing the efficacy of the second-generation irreversible EGFR-TKIs against the first-generation inhibitors, in the second-line setting in advanced squamous NSCLC (LuxLung 8, afatinib vs. erlotinib, www.clinicaltrials.gov: NCT01523587) and in the first-line treatment of patients with EGFR-mutant disease (ARCHER 1050, dacomitinib vs. gefitinib, www.clinicaltrials.gov: NCT01774721). Disappointingly, the results of another phase III trial comparing first and second-generation EGFR-TKIs in the second/third-line setting (ARCHER 1009: dacomitinib vs. erlotinib) do not support the greater clinical efficacy of the newer EGFR-TKIs in unselected patients with advanced NSCLC (55). However, the results from the two on-going phase III trials, mentioned above, are eagerly awaited.

### Third-generation EGFR-TKIs and newer recombinant anti-EGFR monoclonal antibodies

A number of third-generation EGFR-TKIs are also in development, with AZ9291 and CO-1686 being the most advanced of these T790M-specific small molecule inhibitors. Results of early phase I trials of both AZ9291 and CO-1686 in EGFR mutation-positive NSCLC patients previously treated with EGFR-TKIs are encouraging. In both trials, which required screening biopsies for centralized T790M mutation testing, objectives responses of ~60% in patients testing T790M positive at screening have been observed (56, 57), with responses also noted in patients lacking the T790M mutation receiving AZ9291, albeit less frequently (23%) (56). Definitive phase III trials of these molecularly targeted agents are planned.

The recent success of the newer recombinant anti-EGFR monoclonal antibody, necitumumab, in combination with gemcitabine–cisplatin chemotherapy over chemotherapy alone in the first-line treatment of patients with advanced squamous NSCLC (58) (OS: 11.5 vs. 9.9, *p* = 0.012) is also of interest in advanced NSCLC. Although potentially effective in the primary resistance setting, what role necitumumab will play in the acquired resistance setting in patients with squamous and non-squamous NSCLC histology remains to be determined.

### Dual targeting of the EGFR kinase domain

Dual targeting of the EGFR kinase domain, although historically quite toxic, will also likely continue to be explored as a strategy to optimize EGFR-TKI therapy given the phase I dose-escalation and expansion trial demonstrating ORR of ~30% with dual therapy with the anti-EGFR monoclonal antibody cetuximab and afatinib in patients with acquired resistance to first-generation EGFR-TKIs (59). These encouraging results, however, are tempered by the continued toxicity of this combination regimen with 77 and 69% of patients experiencing rash and diarrhea (any grade), respectively (59).

### Second-generation ALK inhibitors

As secondary mutations in the ALK domain have been identified in approximately one third of patients with acquired resistance to the ALK inhibitor crizotinib (60), clinical trials in the ALK resistance setting are also focused on the evaluation of more potent second-generation molecular therapies. Of note, high response rates with the newer second-generation ALK inhibitor, ceretinib, have been observed in patients with and without secondary ALK mutations (54), suggesting that their benefit in ALK-resistant disease may not be limited to patients with secondary ALK mutations. A number of other second-generation ALK inhibitors are also in early clinical development (e.g., AP26113), with promising phase I/II results emerging (61).

### Dual EGFR and MET inhibition

While the dual inhibition of EGFR and MET-mediated pathway inhibition by direct MET targeting has not been successful to date, other c-MET inhibitors are being investigated (e.g., INC 280, XL184). A related mechanism of EGFT-TKI resistance is MET activation by its ligand hepatocyte growth factor (HGF) (62) that when overexpressed enables this compensatory mechanism of pathway activation. Preliminary results of a phase I clinical trial evaluating dual inhibition of EGFR and HGF with erlotinib and rilotumumab (AMG 102), a HGF-binding monoclonal antibody, have recently been reported (63) and a phase II study is on-going.

### EGFR and HSP 90 inhibitors

As a molecular chaperone for proteins involved in MET, HGF, and EML4/ALK fusion, the inhibition of heat shock protein 90 (HSP90) is also being considered as a target for compensatory pathway inhibition and has fueled combination therapies in both EGFR-TKI and ALK inhibitor-resistant disease. In the advanced NSCLC EGFR-TKI resistant setting, the HSP90 small molecule inhibitor AUY922 is being evaluated as monotherapy in a phase II clinical trial vs. pemetrexed or docetaxel in patients with tumors with activating EGFR mutations (www.clinicaltrials.gov: NCT016461250). This agent is also being assessed in a phase II trial in patients with *de novo* resistant T790M mutations not previously treated with EGFR-TKIs (www.clinicaltrials.gov: NCT01854034) and in patients with EGFR mutations and/or EGFR-TKI resistant disease, as part of a phase II cluster study in Chinese patients evaluating five novel inhibitors of HSP90, PI3K, ALK, MET, and MEK (64). Further, AUY922 is also being assessed in combination with erlotinib in patients who have previously responded to EGFR-TKIs and/or whose tumors harbor activating EGFR mutations (www.clinicaltrials.gov: NCT01259089), with results expected in the near future. The safety and activity of another HSP90 inhibitor, ganetespib (STA-9090), has also been assessed in a heavily pre-treated population with NSCLC in a phase II single arm trial with three cohorts (EGFR^+^, KRAS^+^, EGFR/KRAS wild-type) (65). In this study, partial responses were noted in 4/66 patients in the EGFR/KRAS wild-type cohort, all of whom were retrospectively confirmed to have disease that harbored the ALK gene rearrangement (65). Despite interest in this HSP90 inhibitor in combination with chemotherapy (GALAXY-1, GALAXY-2) (66, 67), ganetespib’s role in inhibiting EGFR is unclear. Given encouraging preclinical data in ALK-driven tumors resistant to crizotinib (68), ganetespib is being investigated in clinical trials in NSCLC patients with ALK-driven tumors, as a monotherapy in heavily treated (crizotinib naïve) patients (www.clinicaltrials.gov: NCT01562015) and in combination with crizotinib in patients with prior exposure to crizotinib (www.clinicaltrials.gov: NCT01579994).

## Conclusion

Over the last decade, our understanding of the EGF receptor and our ability to target it has evolved significantly, from single receptor first-generation inhibitors in unselected populations to biomarker-driven clinical trials of more potent second and third-generation irreversible multi-targeted EGFR-TKIs and humanized monoclonal antibodies. The failure of earlier trials targeting the EGF receptor was in part due to the lack of good predictive biomarkers of efficacy. The future success of targeted strategies addressing resistance will hinge on our ability to identify these biomarkers and selectively enroll patients to clinical trials, a strategy that has been more successfully applied in the approval of ALK inhibitors. Furthermore, in order to be successful in the acquired resistance setting, rebiopsy, and tailored mechanism-driven strategies will be required at the time of progression, with a concurrent reduction in the toxicity of multi-targeted and combination therapies. Importantly, the knowledge gained from investigations of EGFR and ALK inhibition over the last decade can be applied to the testing of novel therapies targeting newly discovered oncogenic drivers in NSCLC (69) in order to optimize study designs and streamline regulatory approval, to the benefit of all patients with NSCLC.

## Conflict of Interest Statement

The authors declare that the research was conducted in the absence of any commercial or financial relationships that could be construed as a potential conflict of interest.
